# An Introductory Lecture on Pictorial Anatomy

**Published:** 1842-10

**Authors:** 


Art. III.—*An Introductory Lecture on Pictorial Anatomy, delivered to the Students of the School of Design of the Honorable the Commissioners of the Board of Trustees for the Encouragement of Scottish Manufacturers.* By James Miller, F.R.S.E. F.R.C.S.E.—*Edinburgh,* 1842. 8vo, pp. 32.

Mr. Miller's Lecture, which has been published at the request of the Board of Trustees for the encouragement of Scottish manufactures, is an eloquent vindication of the utility of anatomy, as applied to the plastic arts, conceived in good taste, and on the whole most judiciously executed. Were we to suggest a defect, it would be that Mr. Miller in demonstrating the *use* has neglected to warn the artist against the *abuse* of anatomical knowledge; a caution by no means superfluous, when we recollect that such artists as Donatello, John of Bologna, and Fuseli made shipwreck on this shoal, and that Buonarotti himself might have left even a greater name, had he made less prominent display of his anatomical knowledge. Anatomy is but one of the many auxiliaries requisite for the painter and sculptor, and none of them perhaps require more cautious and skilful treatment, greater judgment, or more chastened taste to enable the artist to fully avail himself of their help, without falling into exaggerations, almost as painful, in an artistic sense, as deformity itself. Art is most triumphant when its efforts are least conspicuous, and of all the resources of art, a knowledge of anatomy, we mean the anatomy of external form, is at once the most indispensable, and that which should be the least ostentatiously displayed, and the most carefully subdued, so that its influence should be felt rather than seen.

Mr. Miller's observations on the utility of anatomy in depicting the more minute details of the human form, are very happy and judicious. He instances the "Elgin marbles, where the details of the subordinate parts, the loose hanging folds of the skin, the veins under the belly or on the sides of the horses, more or less swelled as the animal is more or less in action, are given with scrupulous exactness, almost resembling casts taken from the life. And yet who ventures to say that these are not replete with beauty and grandeur?" John Bell, (Observations on Italy, p. 330,) himself a master in anatomical science and pictorial art, but no very warm advocate of anatomical sculpture and painting, has noticed a beautiful example of this anatomy of detail in the dying gladiator, perhaps, take it all in all, the most glorious and exquisite relic of ancient art. He says: "No affectation of anatomy here, not a muscle to be distinguished, a full fleshy skin covers all the body, yet the general forms perfect as if they were expressed. The only anatomical feature discernible is that of full turgid veins, yet not ostentatiously protruded, but seen slightly along the front of the arms and ancles, giving, like the clotted hair, proof of violent exertion."

But we must conclude, or the fascination of the subject, rendered still more seductive by the admirable manner in which Mr. Miller has handled it, will lead us to transgress all reasonable limits. Mr. Miller is evidently endowed with a vivid but pure sense of artistic beauty, and every lover of the fine arts must peruse his lecture with both pleasure and advantage.

